# Correlation of BMI With Dynamic Pulmonary Function Indices in Nonobese Young Adults: A Cross-Sectional Study

**DOI:** 10.7759/cureus.114579

**Published:** 2026-08-15

**Authors:** Archana Kumari, Mary P Bara, Mani Bhushan K Sinha, Rajesh K Sinha

**Affiliations:** 1 Physiology, Rajendra Institute of Medical Sciences, Ranchi, IND

**Keywords:** adolescents, body mass index, obesity, pulmonary function, spirometry

## Abstract

Background and aim

Pulmonary function reflects the functional capacity of the lungs, airways, thoracic cage, and respiratory muscles. BMI, a widely used indicator of nutritional status, has been extensively studied for its association with pulmonary function. However, this relationship remains inconsistent in nonobese individuals, in whom BMI may have only a limited influence. This study aimed to evaluate the relationship between BMI and dynamic pulmonary function indices in healthy nonobese young adults.

Methods

This cross-sectional analytical study was conducted in the Department of Physiology, Rajendra Institute of Medical Sciences, Ranchi, India, from December 2024 to December 2025. A total of 162 apparently healthy young adults aged 18-25 years with BMI <25 kg/m² were included. Pulmonary function tests were performed using a computerized spirometer in accordance with the American Thoracic Society guidelines. Pulmonary function parameters were compared across BMI categories. Pearson’s correlation analysis was used to assess the relationship between anthropometric parameters and pulmonary function indices. Multivariable linear regression analyses were performed to identify independent predictors of forced vital capacity (FVC) and forced expiratory volume in one second (FEV₁).

Results

No statistically significant differences in pulmonary function parameters were observed across BMI categories (all p > 0.05). Height demonstrated a strong positive correlation with FVC (r = 0.608) and FEV₁ (r = 0.637), while body weight showed moderate positive correlations with FVC (r = 0.308) and FEV₁ (r = 0.287) (all p < 0.01). BMI was not significantly correlated with any spirometric parameter. In multivariable regression analyses, height and sex were significant independent predictors of both FVC and FEV₁, whereas BMI and age were not independently associated with either outcome.

Conclusions

Among healthy nonobese young adults, pulmonary function parameters did not differ significantly across BMI categories, and BMI was not independently associated with spirometric indices. Height showed the strongest independent association with FVC and FEV₁, while sex also contributed significantly after adjustment for age and BMI. These findings suggest that body size, particularly height, has a greater influence on lung volumes than BMI within the nonobese range.

## Introduction

Pulmonary function represents the combined activity of the lungs, airways, thoracic cage, and respiratory musculature. Spirometry is the most commonly used method for respiratory assessment, and forced vital capacity (FVC), forced expiratory volume in one second (FEV₁), FEV₁/FVC ratio, peak expiratory flow rate (PEFR), and mid-expiratory flow rates are objective measurements. These indices are also important in the evaluation of ventilatory capacity and comparison of ventilatory function among apparently normal individuals, since age, sex, and body size are important determinants of lung volumes and are used in reference equations [1].

A commonly used measure of nutritional status, BMI, has been studied for its effects on pulmonary function. Results indicate that low and high BMI can affect respiratory physiology through effects on respiratory muscle strength, chest wall compliance, and diaphragmatic mobility [2,3]. Mechanically, in obese people, excessive fat tissue can restrict lung expansion [2].

Longitudinal studies indicate that weight gain is a predictor of progressive loss of lung function over time [4,5]. BMI has not, however, consistently identified whether loss of lean body mass or fat redistribution is responsible for changes in pulmonary mechanics, because these two factors have different effects [2,4]. Other anthropometric variables, such as height, have shown more consistent and stronger correlations with lung volume variables such as FVC and FEV₁ [3,6,7]. Lung function is more likely to reach its maximum in early adulthood; thus, it is critical to understand the determinants of lung function in this group for accurate clinical interpretation. Hence, we chose to perform our study on the relationship between BMI and dynamic pulmonary function indices in nonobese young adults. The major aim was to evaluate the correlation between BMI and spirometric parameters. Secondary objectives were to investigate the correlation of height and weight with pulmonary function, assess differences in pulmonary function parameters between BMI groups, and determine independent predictors using multivariable regression analysis.

## Materials and methods

Study design and setting

This cross-sectional analytical study was conducted in the Department of Physiology at a tertiary care teaching hospital in Ranchi, India, from December 2024 to December 2025.

Ethical approval

The study was approved by the Institutional Ethics Committee (IEC reg. no. ECR/769/INST/JH/2015/RR-21; approval no. 390; Date: December 2, 2024). Written informed consent was obtained from all participants, and confidentiality was maintained throughout the study.

Study population

A total of 162 apparently healthy young adults (medical students) aged 18-25 years were included using convenience sampling. Nonsmokers without any known systemic illness were eligible for inclusion. Participants with respiratory diseases (asthma, chronic obstructive pulmonary disease, or tuberculosis), cardiovascular diseases, recent acute illness, smoking or substance use, pre-obesity or obesity (BMI ≥25 kg/m²), or any condition affecting pulmonary function were excluded.

Sample size

The required sample size was calculated using the following formula:

\(n = \left(\frac{Z_{1-\alpha/2} + Z_{1-\beta}} {0.5 \times \ln\left(\frac{1+r}{1-r}\right)}\right)^2 + 3,\)

where r represents the expected correlation coefficient, Z(1-α/2) corresponds to the standard normal deviate at a 5% significance level (1.96), and Z(1-β) corresponds to 80% power (0.84). Assuming an anticipated correlation coefficient of 0.30 based on previous studies [8], the minimum required sample size was estimated to be approximately 85 participants. To improve statistical robustness and allow subgroup analyses, a total of 162 participants were included in the study.

Data collection and anthropometric measurements

Baseline demographic data were collected using a standardized semi-structured questionnaire. Height was measured using a stadiometer, and weight was measured using a calibrated digital weighing scale (accuracy, 0.1 kg) with participants standing barefoot. Three measurements were obtained for each parameter, and the mean values were used for analysis. BMI was calculated as weight (kg)/height² (m²) and categorized as underweight (<18.5 kg/m²), normal (18.5-22.9 kg/m²), and overweight (23-24.9 kg/m²).

Pulmonary function testing

Pulmonary function tests were performed with participants in the sitting position using a nose clip and a computerized spirometer (RMS Helios 401; Recorders & Medicare Systems, Panchkula, India), following the American Thoracic Society guidelines. Participants were instructed to take a deep inspiration followed by a forceful expiration. The best values (maximum FVC and FEV₁) from at least three acceptable and reproducible maneuvers were used for analysis. The pulmonary function parameters assessed included FVC, FEV₁, FEV₁/FVC ratio, forced expiratory flow (FEF₂₅-₇₅), and PEFR.

Quality control and bias reduction

Measurement bias was minimized through the use of standardized protocols, trained personnel, and calibrated instruments. However, the use of convenience sampling and a student population may have introduced selection bias.

Statistical analysis

Data were coded and entered into Microsoft Excel (Microsoft Corporation, Redmond, WA, USA) and analyzed using IBM SPSS Statistics for Windows, version 25.0 (released 2017; IBM Corp., Armonk, NY, USA). Continuous variables were expressed as mean ± SD. The Shapiro-Wilk test was used to assess normality. One-way ANOVA was performed to compare pulmonary function parameters across BMI groups. Pearson’s correlation coefficient was used to assess relationships between anthropometric variables and pulmonary function parameters. Multivariable linear regression analysis was performed to identify independent predictors of FVC. A p-value <0.05 was considered statistically significant.

## Results

A total of 162 participants were included in the study. The mean age was 21.06 ± 1.99 years. Baseline characteristics are presented in Table 1.

**Table 1:** Baseline characteristics of the study population.

Anthropometric parameters	Mean ± SD
Age (years)	21.06 ± 1.99
Height (cm)	169.12 ± 6.93
Weight (kg)	61.99 ± 10.14
BMI (kg/m²)	21.65 ± 3.82

The study population consisted of 58.6% male participants. The majority of participants were in the normal BMI category (63.0%), followed by the underweight (19.1%) and overweight (17.9%) categories; there were no obese participants (Table 2). The distribution of males and females did not differ significantly across BMI categories (χ² = 3.019, df = 2, p = 0.221; Cramer’s V = 0.137).

**Table 2:** Anthropometric parameters of the study population.

Variable	Subgroup	N (%)
Sex	Male	95 (58.6%)
	Female	67 (41.4%)
	Total	162 (100.0%)
BMI category	Underweight (<18.5 kg/m²)	31 (19.1%)
	Normal (18.5-22.9 kg/m²)	102 (63.0%)
	Overweight (23-24.9 kg/m²)	29 (17.9%)
	Total	162 (100.0%)

Baseline spirometric parameters were within the normal range in all participants. Descriptive statistics for the spirometric variables are presented in Table 3.

**Table 3:** Baseline spirometric parameters of the study population. FEF₂₅-₇₅, forced expiratory flow between 25% and 75% of forced vital capacity; FEV₁, forced expiratory volume in one second; FVC, forced vital capacity; PEFR, peak expiratory flow rate

Spirometric parameter	Mean ± SD
FVC (L)	6.84 ± 0.43
FEV₁ (L)	5.80 ± 0.37
FEV₁/FVC (%)	84.99 ± 5.57
FEF₂₅-₇₅ (L/s)	3.60 ± 0.71
PEFR (L/s)	6.95 ± 1.05

Parametric analyses were used because the spirometric variables were normally distributed (Table 4). There were no significant differences in FVC, FEV₁, FEV₁/FVC ratio, FEF₂₅-₇₅, or PEFR between BMI categories (all p > 0.05) (Table 5).

**Table 4:** Shapiro-Wilk test for normality of spirometric variables. W = Shapiro-Wilk test statistic

Variable	Shapiro-Wilk W	p-value
FVC	0.987	0.137
FEV₁	0.994	0.696
FEV₁/FVC	0.987	0.125
FEF₂₅-₇₅	0.991	0.443
PEFR	0.993	0.686

**Table 5:** Comparison of spirometric parameters across BMI categories. p-values were calculated using one-way ANOVA. FEF₂₅-₇₅, forced expiratory flow between 25% and 75% of forced vital capacity; FEV₁, forced expiratory volume in one second; FVC, forced vital capacity; PEFR, peak expiratory flow rate

Parameter	Underweight (n = 31)	Normal (n = 102)	Overweight (n = 29)	F value	p-value
FVC (L)	6.76 ± 0.43	6.88 ± 0.44	6.80 ± 0.40	1.1	0.491
FEV₁ (L)	5.78 ± 0.38	5.81 ± 0.37	5.78 ± 0.36	1.51	0.277
FEV₁/FVC (%)	85.60 ± 4.45	84.71 ± 5.87	85.37 ± 5.63	2.01	0.143
FEF₂₅-₇₅ (L/s)	3.74 ± 0.68	3.53 ± 0.74	3.69 ± 0.63	1.02	0.548
PEFR (L/s)	6.96 ± 1.03	6.91 ± 1.08	7.05 ± 1.01	0.45	0.97

**Figure 1:**
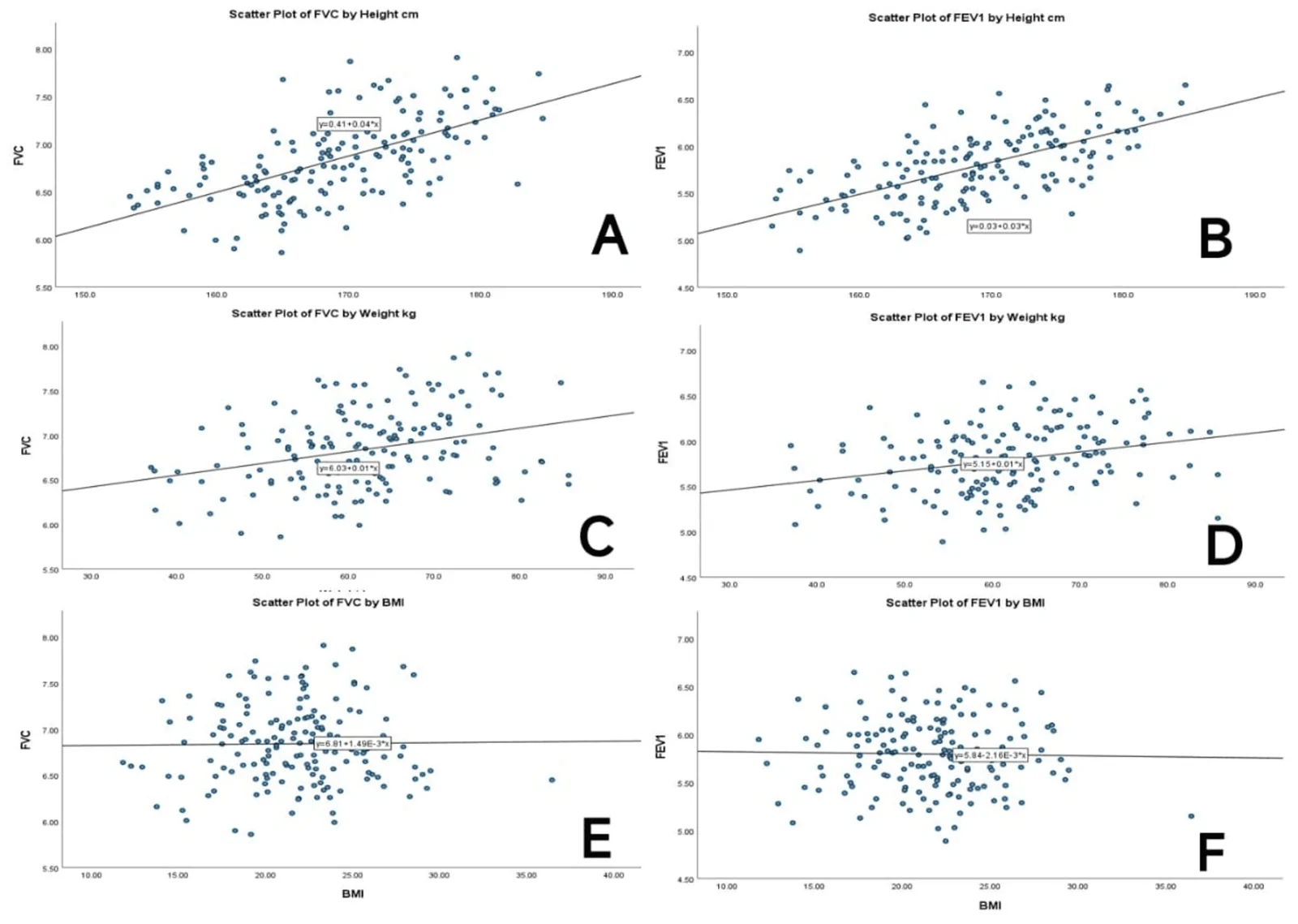
Scatter plots illustrating the relationship between anthropometric parameters and spirometric indices. (A) Height vs. FVC. (B) Height vs. FEV₁. (C) Weight vs. FVC. (D) Weight vs. FEV₁. (E) BMI vs. FVC. (F) BMI vs. FEV₁. Height demonstrated a strong positive correlation with FVC (r = 0.608) and FEV₁ (r = 0.637), while weight showed moderate positive correlations with FVC (r = 0.308) and FEV₁ (r = 0.287). BMI was not significantly correlated with either FVC (r = 0.013) or FEV₁ (r = -0.022). FEV₁, forced expiratory volume in one second; FVC, forced vital capacity

There was a strong positive correlation between height and FVC and FEV₁ and a weaker but still significant positive correlation between body weight and these parameters (Figure 1).

After adjustment for age, sex, and BMI, height remained the strongest independent predictor of both FVC and FEV₁. Sex was independently associated with FVC but not with FEV₁. BMI demonstrated weak positive associations with both spirometric parameters; however, these associations did not reach statistical significance. Age was not independently associated with either outcome. The regression model explained 50.9% and 47.5% of the variability in FVC and FEV₁, respectively, indicating that anthropometric characteristics, particularly height, accounted for a substantial proportion of the observed variation in pulmonary function (Table 6).

**Table 6:** Multivariable linear regression analysis of factors independently associated with FVC and FEV₁. FVC: R = 0.713, R² = 0.509, adjusted R² = 0.496, F = 40.69, p < 0.001; FEV₁: R = 0.689, R² = 0.475, adjusted R² = 0.461, F = 35.28, p < 0.001 FEV₁, forced expiratory volume in one second; FVC, forced vital capacity

Predictor	FVC β	p-value	FEV₁ β	p-value
Age (years)	0.096	0.092	0.000	1.000
Sex	0.169	0.003	-0.108	0.058
Height (cm)	0.758	<0.001	0.802	<0.001
BMI (kg/m²)	0.12	0.076	0.15	0.09

Multicollinearity among independent variables was assessed using tolerance values. Tolerance represents the proportion of variance in a predictor not explained by other variables in the model. All predictors demonstrated acceptable tolerance values (>0.2), indicating the absence of significant multicollinearity. No evidence of problematic multicollinearity was observed among the independent variables, as all tolerance and variance inflation factor values were within acceptable limits (Table 7). The general linear model is presented in Table 8.

**Table 7:** Assessment of multicollinearity in the multivariable regression. Tolerance >0.20 and VIF <5 were considered indicative of the absence of problematic multicollinearity. VIF, variance inflation factor

Predictor	Tolerance	VIF
Age	0.978	1.023
Sex	0.978	1.022
Height	0.786	1.272
BMI	0.786	1.273

**Table 8:** General linear model evaluating determinants of FVC after adjustment for age, height, and sex. R² = 0.488; adjusted R² = 0.465 FVC, forced vital capacity

Source	df	F	p-value
Age	1	1.688	0.196
Height	1	122.988	<0.001
Sex	1	5.588	0.019
BMI category	2	2.565	0.097
Sex × BMI category	2	0.62	0.539

## Discussion

Our study evaluated the association between anthropometric parameters and dynamic pulmonary function indices in healthy young nonobese adults. Spirometric parameters were within the physiological range and were comparable between BMI groups (p > 0.05). The mean FVC and FEV₁ were 6.84 ± 0.43 L and 5.80 ± 0.37 L, respectively, while the FEV₁/FVC ratio (84.99 ± 5.57%) indicated preserved airway patency. BMI was not significantly associated with spirometric parameters in the unadjusted analyses, and this finding remained consistent after adjustment for age, sex, and height. These findings suggest that fluctuations in BMI within the normal range do not significantly influence pulmonary function in healthy young adults [2,9,10].

The high positive correlation between height and lung volumes was one of the main findings of this study. Height correlated significantly with both FVC (r = 0.608) and FEV₁ (r = 0.637) and remained the strongest independent predictor of both parameters after adjustment for age, sex, and BMI (β = 0.758 for FVC and β = 0.802 for FEV₁; p < 0.001 for both). This is consistent with the known relationship between lung volumes, thoracic dimensions, and body height [11,12]. The correlations of body weight with FVC (r = 0.308) and FEV₁ (r = 0.287) were also moderate and positive, as noted by Tang et al. [3], who reported significant differences in spirometric parameters between anthropometric groups. The relevance of body size to lung function was also highlighted by Alam and Samui [10], who found a positive relationship between body weight and dynamic pulmonary function parameters in nonobese young adults. Although BMI demonstrated weak positive associations with FVC and FEV₁ after multivariable adjustment, these associations were not statistically significant. Together with the negligible unadjusted correlations (FVC: r = 0.013; FEV₁: r = -0.022), these findings suggest that BMI alone may not be a reliable predictor of pulmonary function in healthy young adults [12-14].

Our findings are consistent with those of Patel et al. [11], who reported that pulmonary function remained largely preserved among healthy young adults across BMI categories, whereas reductions in FVC and FEV₁ become more evident with increasing adiposity. The mechanical effects of adiposity were probably minimal in the present study because only nonobese individuals (mean BMI: 21.65 ± 3.82 kg/m²) were included [2,14]. Similar observations have been reported in adolescents and young adults, in whom pulmonary function remains largely unaffected within normal BMI ranges [7,9,10]. Walid et al. [7] likewise found no significant differences in spirometric indices between BMI groups, indicating preservation of lung function in nonobese individuals. In contrast, pulmonary function has been shown to decline in overweight and obese populations [2,5,14]. Babu and Gm [13] reported reduced pulmonary function with increasing BMI, whereas PEFR (6.95 ± 1.05 L/s) did not differ significantly between BMI categories (p = 0.97) in the present study, probably because obese participants were not included [13]. Similarly, De Soomer et al. [5] showed that excess adiposity has a negative impact on lung mechanics, especially in overweight and obese adults.

Another possibility for the lack of a significant association between BMI and pulmonary function is that BMI reflects body weight relative to height rather than body composition. BMI does not distinguish between lean body mass and fat distribution, both of which may influence respiratory mechanics differently [2,14,15]. Additionally, the Asian BMI classification cutoffs may influence these associations because they account for regional differences in body composition and metabolic risk. Further evidence of the significance of anthropometric variation comes from population-based data. The varying trends in thinness and obesity in India, as reported by Sung et al. [16], emphasize the need to better understand the physiological consequences of BMI variation.

This study population was relatively young (21.06 ± 1.99 years), an age at which lung function is generally at its physiological peak. Thus, the influence of BMI on pulmonary function may differ in older individuals or those with underlying medical conditions [2,4,5]. Previous studies have consistently demonstrated positive associations between anthropometric measurements and FVC and FEV₁ [3,6,10,12]. Similarly, De Soomer et al. [5] and Naila and Laghari [15] discussed the importance of body size and body composition in relation to pulmonary function, while Sung et al. [16] highlighted the changing anthropometric profile of the Indian population and the need to evaluate BMI-related physiological outcomes.

In our study, the multivariable regression models explained 50.9% of the variability in FVC and 47.5% of the variability in FEV₁, with height remaining the strongest independent predictor of both spirometric parameters. However, the cross-sectional design precludes causal inference. In addition, convenience sampling and the inclusion of a homogeneous group of medical students may have introduced selection bias and limited the generalizability of the findings. BMI was also used as a surrogate measure of body composition and therefore could not distinguish between body fat distribution and lean body mass. Within these limitations, the findings suggest that structural anthropometric characteristics, particularly height, are more strongly associated with pulmonary function than BMI among healthy nonobese young adults. These observations are most applicable to similar healthy young populations and may not be generalizable to older adults or individuals with obesity or chronic disease.

## Conclusions

Our study found no significant differences in spirometric indices across BMI categories among healthy nonobese young adults. Similarly, BMI was not independently associated with pulmonary function after adjustment for age, sex, and height. In contrast, height demonstrated the strongest independent association with both FVC and FEV₁, highlighting the importance of structural anthropometric characteristics in determining pulmonary function. These findings suggest that, within the nonobese range, height is a more important determinant of pulmonary function than BMI. The study emphasizes the importance of considering height and other anthropometric characteristics when interpreting spirometric values in healthy young adults and contributes to a better understanding of the determinants of pulmonary function during early adulthood.
